# Unmasking aflatoxin hazards in maize for human consumption: investigating maize contamination in Mwanza Markets, Tanzania

**DOI:** 10.4314/ahs.v24i3.15

**Published:** 2024-09

**Authors:** Betrand Msemwa, Justine J Mabumbwiga, Caroline A Minja, Shukrani B Phillip, Vitus Silago, Stephen E Mshana, Martha F Mushi

**Affiliations:** 1 Department of Medical Laboratory Sciences, Catholic University of Health and Allied Sciences, Bugando. P.O.Box 1464 Mwanza, Tanzania; 2 Department of Microbiology and Immunology, Catholic University of Health and Allied Sciences, Bugando. P.O.Box 1464 Mwanza, Tanzania; 3 Department of Biochemistry and Molecular biology, Catholic University of Health and Allied Sciences, Bugando. P. O. Box 1464 Mwanza, Tanzania; 4 Institute of Allied Health Sciences, Ruaha Catholic University, P. O. Box 774, Iringa, Tanzania

**Keywords:** Maize vendors, maize contamination, polypropylene woven bags, Aflatoxin

## Abstract

**Background:**

Aflatoxin arises from toxigenic Aspergillus species, which infect maize because of improper storage, insufficient drying, extended storage periods, and suboptimal farming practices. This study investigated the aflatoxin contamination in maize for human consumption within specific markets of Mwanza, Tanzania.

**Methodology:**

A cross-sectional study was conducted from June to August 2021. Maize samples were analyzed using ELISA followed by descriptive statistical data analysis.

**Results:**

A total of 90 maize merchants from 8 local markets were involved. Their mean age was 34.7 (± 6.7) years, majority were male (51 out of 90, 56.7%). Among the vendors, the majority were not aware of aflatoxin (62 out of 90, 68.9%), stored maize in polypropylene woven bags (62 out of 90, 68.98%) and dried the maize before storage (86 out of 90, 95.6%).

Out of the 90 samples, 10 (11.1%) had aflatoxin contamination above 1µg/kg, ranging from 1.01µg/kg to 33.4µg/kg with 3 (3.3%) being contaminated above the acceptable standard (≥10µg/kg).

**Conclusion:**

The levels of aflatoxin contamination in maize for human consumption exceed the established safety thresholds. Governments in lower and middle-income nations should intensify enforcement of regulations aimed at enhancing community awareness regarding aflatoxin risks and minimizing contaminations.

## Background

Aflatoxin is a fungal secondary metabolite produced by some strains of Aspergillus flavus and Aspergillus parasiticus 1, 2. They are commonly found in cereals, milk, tree nuts and oilseeds. There are about 18 types of aflatoxins: B1, B2, G1, G2, M1, M2, P, Q etc. 3. The production of aflatoxin is highly favored by poor conditions of harvesting, transport and storage, either in the farm, in the market or in housing, thus inducing growth of the fungus and the production of secondary metabolites^4^. Aflatoxins B1, B2 and G1, G2 which are blue and green under UV light, respectively, are the commonest produced toxins 5. The toxins are known to be highly toxic, teratogenic, carcinogenic and mutagenic both in human and animals 6. Aflatoxin B1 has been documented to have acute toxicity both in kidney and liver with high stability in different conditions that lead to great economical loss 7. The United Nations Food and Agriculture Organization estimate that up to 30% of food supplies in the world are contaminated with aflatoxin 8. This contamination is estimated to contributes from 4.6% to 28.2% of all liver cancer cases, most of which occur in sub-Saharan Africa, Southeast Asia and China 9. In Tanzania, the maximum allowed limit of aflatoxin contamination in food product is 10 µg/kg 10, however, the study conducted in the rural settings of the country reported up to 18% of maize harvested being contaminated by up to 158 µg/kg of aflatoxins^11^. Furthermore, in Tanzania, 40% of children and infants are reported to be exposed to aflatoxins through diet^12^. Mwanza is the second most populated city in Tanzania after Dar es Salaam. Just like in other regions, in the city of Mwanza maize is the primary dietary staple and main crop produced by small scale farming 13. Most of the maize produced is stored at household level for food consumption in the family or selling to cater for other family needs. In the maize market, vendors collect small portion from different farmers and redistribute. In the market vendors store maize in different facilities include Polypropylene bags, Polythene and plastic bags which are documented to be prone to maize contamination by aflatoxin producing fungi 14. In Mwanza, Tanzania, the level of aflatoxin contamination in maize is not well known despite the practice towards transportation and maize storage to influence the fungi contamination. Therefore, this work determined the aflatoxin contamination in maize from selected markets in the city of Mwanza, Tanzania, with detail descriptions of the vendors with contaminated maize.

## Material and methods

Descriptive cross-sectional study was conducted from June to August 2021. This study involved eight main cereal markets in the city of Mwanza: Buhongwa, Kirumba, Mkuyuni, and Bugarika markets in Nyamagana district and Mirongo, Buswelu, Buzuruga, and Pansiansi markets in Ilemela district.

### Sample size and sampling procedure

The sample size was determined by considering the number of vendors in each market. This approach guarantees a precise representation of the various sources and varieties of maize available in the market. To ensure fairness in representing vendors across different city markets, a stratified sampling method was employed. The vendors were grouped into homogeneous strata based on the number of vendors present in each specified market as indicated in table 1. In each market/strata the vendors were randomly sampled to reach allocated number.

**Table 1:** Sample size distribution per market

Market	Number of vendors	Collected maize sample
Buhongwa	22	20
Kirumba	10	9
Buswelu	11	10
Buzuruga	11	10
Mirongo	14	13
Bugarika	11	10
Pansiansi	11	10
Mkuyuni	9	8

Predesigned questionnaire was used to collect social-demographic data, maize source, stock size, storage method and awareness on aflatoxin from the maize vendors. A total of 50 grams of maize were measured and collected from each vendor involved in the study into a sterile dry zipper bag and transported on the same day of collection to the Microbiology research laboratory of the Catholic University of Health and Allied Sciences for processing.

**Sample preparation:** In the laboratory maize were processed according to test kit manufacturer instructions (Sigma Aldrich, Saint Louis, MO 63103, USA). Briefly, 2gram of maize sample was measured and introduced into a 50ml tube, 5ml of 70% methanol was added and mixed well by vigorous shaking in 10 inversions, and the mixture was centrifuged at 4000rpm for 10min at room temperature. Thereafter 0.5 ml of supernatant was harvested. Finally, 0.5 ml of deionized water was added and mixed with 0.5 ml of supernatant ready for ELISA test following manufacturer instruction (Sigma Aldrich, Saint Louis, MO 63103, USA).

The competitive ELISA technique was employed to identify aflatoxin presence in maize samples. In this process, a microplate coated with antigens was used. These antigens competed with the antigen in the sample or standard for binding to the HRP-conjugated antibody. Subsequently, a tetramethylbenzidine (TMB) substrate was introduced to induce color development. Optical density (OD) values were then measured for both samples and standards using an ELISA reader. By plotting a standard curve correlating OD with concentration, the aflatoxin concentration was determined. Samples registering an aflatoxin concentration of 1µg/kg or higher were classified as positive for aflatoxin contamination^15^,^16^.

### Ethical considerations

The protocol for conducting this study was reviewed and ethically approved by the Joint CUHAS/BMC ethics and review committee with certificate number 1860/2021. All laboratory experiments forllowed the microbiology standrd operationg procedures. All the maize vendors signed the written informed concent form before being involved in the study.

## Results

### Demographic characteristics of maize merchants

The mean (±SD) age of the maize vendors was 34.7 (±6.7) years, predominantly being male 51(56.7%). A total of 53(58.9%) vendors had primary education as the highest level. Majority of vendors were not aware of aflatoxin 62(68.9%), store maize in polypropylene woven bags 62(68.98%) and dry the maize before storage 86(95.6%). A total of 8(8.9%) vendors stored the maize for more than 6 months (180 days), table 2.

**Table 2:** Social demographic characteristics of the participants

Variable	Category	Frequency(n)	Percentage
**Gender**	Female	39	43.3
	Male	51	56.7
**Education level**	Primary	53	58.9
	Secondary	37	41.1
**Source of maize**	Farmers	37	41.1
	Merchants	53	58.9
**Stock size (bags)**	1 to 10	53	58.9
	10 to 50	35	38.9
	50 to 100	2	2.2
**Aflation awareness**	No	62	68.9
	Yes	28	31.1
**Maize storage places**	Home	27	30
	Market	63	70
**Type of storage bag**	Plastic	5	5.6
	Polypropylene woven	62	68.9
	Maize	1	1.1
	Other	22	24.4
**Pesticide use**	Yes	22	24.4
	No	68	75.6
**Drying before storage**	No	4	4.4
	Yes	86	95.6
**Storage duration**	Below 90 days	44	48.9
	90 days	38	42.2
	180 days	8	8.9

### Aflatoxin contaminations

In various markets, out of a total of 90 samples, 10 (11.1%) displayed aflatoxin contamination levels exceeding 1µg/kg, ranging from 1.01 µg/kg to 33.4 µg/kg. The mean concentration of aflatoxin in the contaminated maize was 13.5µg/kg, with a STD of 5.7 µg/kg. Out of the 90 samples, 3 (3.3%) of them surpassed the country's acceptable standard of 10µg/kg. These samples were from two different market with aflatoxin concentrations of 28.6µg/kg, 33.4µg/kg, and 32.1µg/kg, respectively.

For vendors associated with contaminated maize exceeding 1µg/kg, the average age was 34.4 years, with a standard deviation of 5.6 years. Out of the 10 vendors, 3 were female. All vendors with contaminated maize reported the practice of drying the maize before storage (Table 3).

**Table 3:** Detail description of the 10 sample with aflatoxin contamination above 1µg/kg

S/No	Markets	Age	Sex	Source	Bags stocked	Place for storage	Type of storage bag	Pesticide use	Drying before storage	Stock duration	Aflatoxin awareness	aflatoxin content (µg/kg)
1	Kirumba	41	Female	Farmers	10 to 50	Home	Polypropylene woven bags	Yes	Yes	90 days	Yes	2.2
2	Buswelu	29	Female	Vendors	1 to 10	Market	Polypropylene woven bags	No	Yes	90 days	No	1.1
3	Mirongo	29	Male	Vendors	1 to 10	Market	Polypropylene woven bags	No	Yes	Below 90 days	No	32.1
4	Bugarika	37	Male	Vendors	1 to 10	Market	Polypropylene woven bags	No	Yes	Below 90 days	Yes	1.01
5	Bugarika	42	Male	Vendors	1 to 10	Market	other bags	No	Yes	Below 90 days	No	1.8
6	Buhongwa	28	Male	Vendors	1 to 10	Market	Polypropylene woven bags	No	Yes	Below 90 days	No	2.3
7	Mkuyuni	37	Male	Vendors	1 to 10	Market	Polypropylene woven bags	No	Yes	Below 90 days	No	2.3
8	Mkuyuni	40	Female	Farmers	10 to 50	Home	other bags	Yes	Yes	180 days	No	5.0
9	Buzuruga	32	Male	Farmers	10 to 50	Market	Plastic bags	Yes	Yes	90 days	Yes	33.4
10	Buzuruga	29	Male	Farmers	10 to 50	Home	Polypropylene woven bags	No	Yes	90 days	Yes	28.6

## Discussion

Maize contaminations by aflatoxin is of public health importance since it is a staple food and also used as ingredient for most of complementary food in the country 17. This study documented up to 11.1% of maize sampled were contaminated with aflatoxin from 1µg/kg and above. Although this is below the country acceptable standard which is 10 µg/kg it is alerting the possibility of increase contamination with longer storage as documented in previous studies 11, 17. A previous study conducted in India reported increase concentration of aflatoxin with increase days of storage when the moisture level is above 12% 18.

In the current study, the concentration of aflatoxin contaminating maize was found to range from 1.01 µg/kg to 33.4 µg/kg with mean concentration of 13.5(5.7) µg/kg which is above the maximum tolerable limit of 10 µg/kg in Eastern Africa 10 https://www.aflatoxinpartnership.org/wp-content/uploads/2020/06/Tanzania_Aflatoxin_Control_MAY15.pdf. This mean concentration is similar to 13.1 µg/kg previously reported by a study conducted in Kibaigwa an international maize market located in Dodoma Tanzania 17. Duration of storagof maize to more than 90 days was also highlighted in Dodoma study as one of the major factors associated with aflatoxin contamination in maize 17. This finding alerts the possibility of cumulative consumption of low doses of aflatoxin through contaminated maize intake which predisposes consumers to chronic aflatoxicosis 19. The reported mean is lower compared to 20 µg/kg up to 100 µg/kg from a study conducted in four districts in Kenya during the aflatoxicosis outbreak of the year 2004 1. Maize have been pointed out as one of the food which is most frequently contaminated with aflatoxin ad fumonisins from different fungi species pre and post-harvest 20. The annual consumption of maize is estimated to exceed 3 million metric tons in the country with daily per capital consumption of 450g for people in rural settings^21^. Majority of dwellers of Mwanza town consume maize from vendors in local markets involve in the study.

In the current study, the use of wrong storage bags (polypropylene woven bags) was documented more among vendors with contaminated maize. The similar findings was pointed out in another study conducted in Dar es salaam which reported 69 spice samples (57.5%) out of 120 spice samples stored in polypropylene woven bags being contaminated with aflatoxins 22. Polypropylene woven bags are neither moisture nor insect resistant, thus causing maize grains to be very susceptible to moisture and fungi infestation 23. Furthermore, in all surveyed markets no merchants stored maize in Purdue Improved Crop Storage bags (PICS) which is highly recommended. PICS are triple-layer hermetic bags that have been adopted by farmers due to their exceptional ability to control insect pests in cowpea, maize, and peanut 24. Purdue Improved Crop Storage bag use two liners of high-density polyethylene (HDPE) and an outer layer composed of woven polypropylene. Moreover, post-harvest aflatoxin contamination can occur when grain is improperly managed through faulty drying and storage processes under humidity and temperature levels favorable for mold proliferation 25.

## Limitation

The maize samples analyzed in this study were specifically sourced from the top layer of the storage bag. This approach could potentially result in an underestimation of the actual prevalence of aflatoxin contamination in maize intended for human consumption, as there may be disparities in air circulation and moisture distribution within the bag which enhance fungi growth. However, this method was employed to faithfully represent the maize consumed by the community, as vendors typically sell maize from the uppermost layer.

## Conclusion

Aflatoxin contamination exceeding the permissible limit has been identified in maize intended for direct human consumption in Mwanza, Tanzania. It is imperative that the government intensifies its regulatory enforcement efforts to enhance community awareness about aflatoxin and to mitigate contamination risks. Comprehensive measures, encompassing both pre-harvest and post-harvest stages, should be implemented to prevent aflatoxin contamination in maize. Providing farmers and merchants with essential knowledge about aflatoxins and their associated impacts is crucial. Furthermore, it is advisable to conduct additional research to ascertain the levels of aflatoxin exposure among consumers of this maize.

## Data Availability

All data have been included in the manual script.

## List of Abbreviations

AF: Aflatoxin
AFB: Aflatoxin B
AFG: Aflatoxin G
CUHAS: Catholic University of Health and Allied Sciences
ELISA: Enzyme linked Immunosorbent Assay
HRP: Horseradish Peroxidase
PICS: Purdue Improved Crop Storage bags
